# Complete protection against cryodamage of cryopreserved whole bovine and human ovaries using DMSO as a cryoprotectant

**DOI:** 10.1007/s10815-017-0963-x

**Published:** 2017-06-03

**Authors:** Johan R. Westphal, Renne Gerritse, Didi D. M. Braat, Catharina C. M. Beerendonk, Ronald Peek

**Affiliations:** Department of Obstetrics and Gynecology, Radboud University Medical Center, PO Box 9101, 6500 HB Nijmegen, The Netherlands

**Keywords:** Whole ovary, Cryopreservation, Ischemia, Bovine, Human

## Abstract

**Purpose:**

This study aims to determine the optimal cryopreservation protocol for whole ovaries intended for preservation of fertility in women.

**Methods:**

We investigated the optimal cryopreservation procedure for whole ovaries in a bovine model. The following parameters were investigated to determine their effect on ovarian tissue viability: type of cryoprotectant, administration route of the cryoprotectant (perfusion and/or submersion), and the maximum tolerable interval between death of the animal and start of the cryopreservation process. The resulting optimal cryopreservation procedure for bovine ovaries was subsequently tested on human ovaries. In vitro glucose uptake, histology, and immunohistochemistry were used to assess the integrity of the ovarian tissue.

**Results:**

Starting the cryopreservation procedure (including perfusion with and submersion in DMSO) within 10–15 min after death of the animal proved critical, resulting in a 90–100% protection level against cryodamage. When cryopreserving human ovaries using the same protocol, over 95% protection against cryodamage was observed on all tissue levels. In addition, no apparent morphological damage to either the follicles or the vascular endothelium was observed.

**Conclusion:**

Our findings suggest that using the optimized protocol presented in this paper allows good cryopreservation of whole human ovaries and represents an important step in considering whole ovary autotransplantation for clinically applied fertility preservation.

## Introduction

With treatment modalities improving over the years, the number of cancer patients that survive their disease is increasing. Parallel to this encouraging development, growing attention is being given to the quality of life after cure, including the ability to start a family. As some anti-cancer therapies may reduce fertility or even lead to sterility, fertility preservation should be offered prior to the start of these types of therapy. A number of options for fertility preservations are currently available to female patients [1–5]. Depending on age, the type and stage of the cancer, and the stage of life, the most suitable option will have to be selected for each patient individually.

Cryopreservation of cortical ovarian tissue strips is one option for fertility preservation. These tissue strips can, after the patient has been cured of her disease, be thawed and autotransplanted to restore fertility. To date, this option for fertility preservation has already led to the birth of at least 86 children worldwide, indicating the viability of this option [6–8].

The major problem occurring when retransplanting ovarian cortical tissue is the occurrence of ischemia [9]. After surgery, it takes about a week for the strip(s) to become sufficiently vascularized. During this period, many follicles in the graft are lost due to ischemia, thereby limiting the time the graft functions physiologically. The life span of a graft ranges between several months up to 7 years [10–15], after which subsequent autotransplantations may be required to restore fertility.

An alternative for the cryopreservation of ovarian cortical strips is the cryopreservation and subsequent autotransplantation of a whole ovary including the vascular pedicle [16–20]. As the vascular pedicle of the ovary is surgically attached to the circulation, this will result in an immediate restoration of the oxygen and nutrient supply to the graft. Consequently, ischemic damage will be kept to a minimum.

The successful cryopreservation and subsequent thawing of an organ the size of an whole human ovary, however, is no small feat. Proof of concept of the procedure has been provided by Imhof et al. [17] and others [19–22], who were able to cryopreserve and retransplant an intact ovine ovary. However, as an ovine ovary is considerably smaller than a human ovary [23], the cryopreservation of a human ovary has proven to be technically much more complicated. The delivery of sufficient amounts of cryoprotectant to the entire ovary will prove to be challenging. In addition, the requirements for protection against cryodamage may actually differ for the different cellular components of the ovary, i.e., the follicles/oocytes, the stromal cell compartment, and the vascular bed. Bedaiwy et al. [21] and Martinez-Madrid et al. [24, 25] have both made a first attempt at cryopreserving intact human ovaries by perfusing them with a 10% DMSO solution. In these studies, cryodamage of the ovarian tissue was assessed by viability tests, electron microscopy, and analysis of cells undergoing apoptosis, indicating minor damage to the tissue.

For the development of an optimal cryopreservation procedure, it is not possible to use large numbers of human ovaries for both ethical and practical reasons. We therefore initially resorted to a bovine animal model, as these ovaries are very similar to human ovaries in several aspects [23]. Our results may expedite the application of whole ovary cryopreservation and autotransplantation to clinical use.

## Materials and methods

### Experimental design

We collected bovine and human intact ovaries and subjected these ovaries to different cryopreservation protocols. The level of protection against cryodamage that was achieved by applying these different protocols was assessed by three different assays. We used a glucose uptake assay to determine the viability of stromal ovarian tissue (human and bovine tissue), and histological analysis was performed to assess the condition of follicles (human and bovine tissue) and immunohistochemistry (human tissue only) to determine the integrity of the ovarian vasculature.

Using these techniques, we tested the effect on the degree of protection against cryodamage in frozen and thawed intact bovine ovaries in four separate and subsequent sets of experiments (A–D):

A. Submersion in different types of cryoprotectant prior to cryopreservation (outlined in Table 1); intact bovine ovaries were submerged in a 10% solution of four different cryoprotectants for at least 15 min

**Table 1:** Experimental design (Fig. 1)

Condition	Number of ovaries	Submersion	Perfusion	Cryoprotectant	Concentration (%)	Time of submersion and/or perfusion (min)
1 (+ control)	6	na	na	na	na	na
2 (− control)	5	−	−	−	−	0
3	5	+	−	DMSO	10	15
4	4	+	−	Propanediol	10	15
5	4	+	−	Ethylene glycol	10	15
6	4	+	−	Butanediol	10	15

*na* not applicable

B. Perfusion of bovine ovaries with cryoprotectant (Table 2); using the best cryoprotectants identified in experiment 1, we tested the additional effect of perfusion with cryoprotectant in combination with submersion in cryoprotectant

**Table 2:** Experimental design (Fig. 2)

Condition	Number of ovaries	Submersion	Perfusion	Cryoprotectant	Concentration (%)	Time of submersion and/or perfusion (min)
1 (+ control)	6	na	na	na	na	na
2 (− control)	5	−	−	−	−	0
3	5	+	−	DMSO	10	15
4	5	+	+	DMSO	10	30
5	3	+	+	Ethylene glycol	10	30

*na* not applicable

C. The duration of the combined submersion and perfusion treatment with bovine ovaries
D. The duration of the ischemic period between death of the animal and start of the cryopreservation process (both in Table 3)

**Table 3:** Experimental design (Fig. 3)

Condition	LI or SI	Number of ovaries	Submersion	Perfusion	Cryoprotectant	Concentration (%)	Time of submersion and/or perfusion (min)
1 (+ control)	LI	10	na	na	na	na	na
	SI	7					
2 (− control)	LI	5	−	−	−	−	−
	SI	5					
3	LI	5	+	+	DMSO	10	30
	SI	6					
4	LI	nd	na	na	na	na	na
	SI	6	+	+	DMSO	10	60
5	LI	5	+	+	DMSO	10	120
	SI	7					

In all experiments, fresh (i.e., not cryopreserved) tissue was used as a positive control, and tissue with experimentally induced maximal cryodamage was used as a negative control

*na* not applicable, *nd* not done, *LI* long period of ischemia, *SI* short period of ischemia

### Collection of bovine ovaries

Intact bovine ovaries were collected at a local abattoir essentially as described previously [23, 26]. Briefly, whole ovaries with their vascular pedicle used for culturing fresh ovarian cortical, subcortical, and medullar biopsies were collected on ice. Ovaries used for cryopreservation experiments were perfused on site with 15 ml of Ringer’s solution (Baxter, Utrecht, The Netherlands) containing 50 IE/ml heparin (Leo Pharma, Breda, The Netherlands) and 2.5% methylene blue (Clinical Pharmacy, Nijmegen, The Netherlands) via the vena ovarica, until a blue discoloration of the tissue was observed (usually within 2–3 min). The time between death of the animal and start of the perfusion was kept to a minimum but was dependent on the workflow in the abattoir (40–50 min). In a separate set of experiments, we were able to obtain the bovine ovaries within 10–15 min after the death of the animal, thus enabling a much quicker start of the onsite perfusion with heparin. The efficiency of the perfusion was monitored by the appearance of blue coloration at the surface of the ovary. After the perfusion, the ovaries were transported on ice to the laboratory for further processing.

### Human ovaries

Six human ovaries were obtained from three different patients opting for preventive gonadectomy (two BRCA1 patients and one patient with breast cancer; aged 45, 41, and 35 years, respectively) from the Streekziekenhuis Koningin Beatrix, Winterswijk (The Netherlands). Informed consent was obtained from each patient. Approval for the procedure was obtained from the local medical ethical committee, provided that the procedure would not interfere with the required diagnostic histology.

One ovary of each patient was used to prepare fresh tissue fragments. From the other ovary (which was to be cryopreserved), the vascular pedicle was dissected, and an 18 GA 1.77, 1.3 × 35 mm cannula (Venflon, Becton Dickinson, Breda, The Netherlands) was inserted in the arteria ovarica. The ovary was perfused for 5 min with a heparin/methylene blue solution as described for the bovine ovaries. The perfusion was performed within 5 min after the extirpation of the ovary. After perfusion, both ovaries were stored on ice and transported to the laboratory.

### Cryopreservation and thawing of intact bovine and human ovaries

For submersion experiments, the bovine ovaries were placed in a bath containing 30 ml of Dulbecco’s modified Eagle’s medium (DMEM; PAA Laboratories, Pasching, Austria)/2% fetal calf serum (FCS; Gibco, Breda, The Netherlands) without any cryoprotective agent (*n* = 5), or in the presence of 10% cryoprotective agent, including dimethyl sulfoxide (DMSO; Sigma-Aldrich, Zwijndrecht, The Netherlands) (*n* = 5), propanediol (*n* = 4), ethylene glycol (*n* = 4), or butanediol (*n* = 4). Submersion time was varied between 15 and 180 min. Subsequently, the ovaries were transferred to a sterile 100-ml container (Deltalab, Barcelona, Spain) containing DMEM/2% FCS with or without 10% of the cryoprotectants mentioned above. The container was then placed into a passive cooling device (5100 Cryo Freezing Container; Nalgene, VWR, Belgium) precooled to 0 °C. Subsequently, the container was transferred to a −80 °C freezer overnight to allow for a gradual decrease in temperature (detailed in Gerritse et al. [26]). The container with the ovary was then submerged in liquid nitrogen and stored for at least 1 week.

Perfusion of the bovine ovaries was performed by inserting a blunt-ended needle (23 g olive tipped cannula curved, Aspen Medical, USA) into the arteria ovarica and secured using a small clamp [23]. All vessels other than the vena ovarica and the arteria ovarica were occluded using clamps. Perfusion time was varied between 15 and 120 min with a solution of DMEM supplemented with 2% FCS, 2.5% methylene blue, and 10% DMSO or ethylene glycol, using a peristaltic STC-521 syringe pump (Terufusion, Tokyo, Japan), at a flow rate of 2.5 ml/min. During perfusion, the bovine ovaries were submerged in the same solution as the perfusion fluid, only without the methylene blue. During perfusion, we observed backflow of the (blue) perfusion fluid from the vein. No leakage of perfusion fluid from the clamped vessels was observed, illustrating the efficiency of the procedure. After the perfusion, the ovaries (with the blunt-ended needle still inserted) were transferred to a sterile 100-ml container (Deltalab, Barcelona, Spain) containing DMEM/2% FCS with or without 10% of the cryoprotectants mentioned above and were frozen as described previously for the submersion experiments.

Extrapolating on our experimental results regarding glucose uptake by bovine ovaries, human ovaries were perfused with, and submerged in, a 10% DMSO solution for 60 min.

Thawing of whole bovine and human ovaries was performed basically as described by Martinez-Madrid et al. [25]. The ovaries were placed in a water bath at 60 °C for approximately 10 min, until most of the ice surrounding the ovary had melted, leaving the ovary still frozen. Next, ovaries were incubated in a water bath at 37 °C for 20 min, until the tissue was completely thawed. Subsequently, the ovary was taken from the container, submerged in 30 ml of DMEM/2% FCS, and reconnected to the peristaltic pump. The cryoprotectant was gradually removed by three subsequent perfusion steps of 10 min each at room temperature with a flow rate of 2.5 ml/min using DMEM/2% FCS with 0.25 M of sucrose, DMEM/2% FCS with 0.1 M of sucrose, and DMEM/2% FCS without sucrose, respectively.

After perfusion, ovarian biopsies were prepared and cultured as described below.

### Preparation and culturing of ovarian biopsies to assess glucose uptake

Uptake of glucose by cultured ovarian tissue fragments was performed essentially as described [26]. Briefly, from either fresh or cryopreserved/thawed ovaries, end-to-end tissue rods were prepared with a 6-mm-diameter biopsy punch (Kai Medical, Solingen, Germany) and divided in two cortex fragments, two subcortex fragments, and two medulla fragments, each 2–3 mm long. Biopsies were transferred to six-well plates (Falcon, Heidelberg, Germany) for culturing. Duplicate wells were prepared for each ovary. Each well contained either three cortical, three subcortical, or three medullar biopsies from the same ovary, in 5 ml of DMEM culture medium supplemented with 10% FCS and 40 μg/ml gentamicin. At day 4, the culture was ended and the glucose content of culture supernatants was determined using a standard blood gas analyzer (Chiron Diagnostics, Bayer, Germany). At the end of the culture period, biopsies were weighed, and glucose uptake was normalized (and expressed) per milligram tissue per hour. Protection levels were expressed as a percentage of glucose uptake by fresh tissue.

### (Immuno)-histochemical staining of ovarian tissue

To highlight blood vessel endothelial cells and follicles, tissue sections of both fresh and cryopreserved/thawed fragments of human ovaries were fixed overnight in phosphate-buffered 4% formaldehyde and embedded in paraffin. Five-micrometer sections were stained with anti-human factor VIII to visualize the vascular endothelium [23].

For the analysis of follicle integrity, 7-μm sections of either bovine or human ovarian cortex were stained with hematoxylin and eosin. To prevent counting a follicle twice, each subsequent section was separated by 100 μm of tissue. The percentage of morphologically normal and degenerated (i.e., showing cytoplasm shrinkage, disorganized granulosa cells, or pyknotic nuclei) primordial, primary, secondary, and antral follicles was determined according to predefined criteria [27], by two independent observers. The mean value is presented. The percentage rather than the total number of follicles was determined as the distribution of follicles in cortex fragments of the same ovary may vary more than 2 orders of magnitude [28, 29]. All sections were examined by conventional light microscopy (×100).

### Statistical analysis

Data were tested for normality using GraphPad Prism (version 5.13) for Windows (GraphPad Software, San Diego, CA, USA) and found to be normal. Two-sided Student’s *t* test was performed using the same software package. *P* < 0.05 was considered to be statistically significant.

## Results

### Protection against cryodamage by different cryoprotective agents

**Fig. 1:**
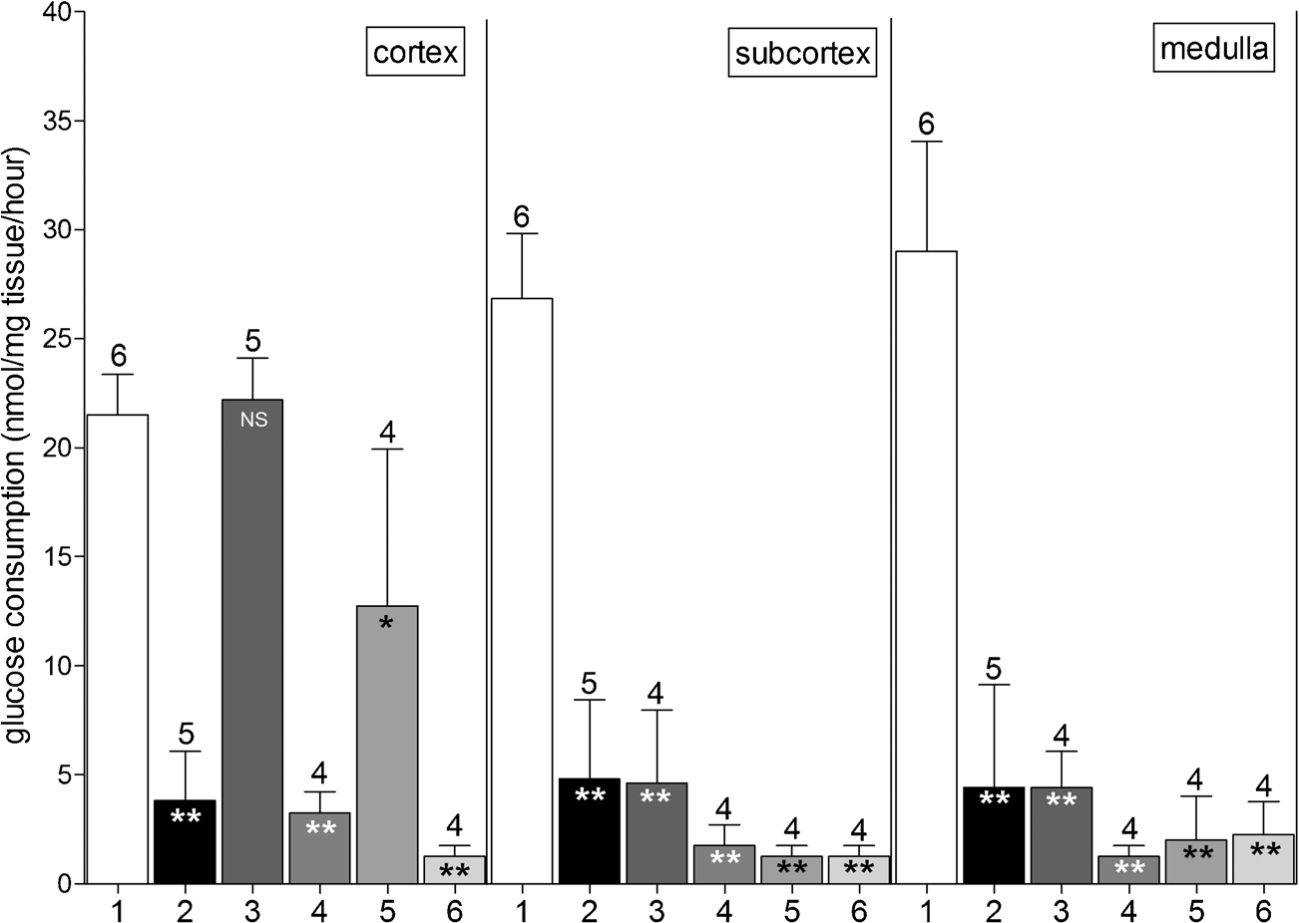
In vitro glucose uptake of ovarian tissue fragments derived from bovine ovaries submerged in cryoprotectants. Fresh ovaries (condition 1; positive control, *white bars*); ovaries cryopreserved and thawed without any protection (condition 2; negative control, *black bars*); from ovaries submerged in a solution containing 10% DMSO (condition 3), propanediol (condition 4), ethylene glycol (condition 5), or butanediol (condition 6) for 15 min prior to cryopreservation. Tissue fragments were collected from the ovarian cortex (*left panel*), the subcortex (*middle panel*), and the medulla (*right panel*). Shown is the mean ±SD; the *number above bars* represents the number of tested ovaries per condition. Statistically significant differences from glucose uptake by fresh tissue are shown in *bars*; *P < 0.05; **P < 0.001; *NS* no statistical significance. Note the complete protection against cryodamage of cortical fragments derived from ovaries submerged in DMSO (condition 3) and the limited protection by submersion in ethylene glycol (condition 5). The other cryoprotectants displayed no protection at all (conditions 4 and 6). In the deeper tissue layers (*middle* and *right panels*), protection against cryodamage by submersion was not observed with any of the cryoprotectants used

In previous work, we have shown that submerging an intact bovine ovary in a 10% DMSO solution for 15 min fully protected the cortical layer of the ovary, while leaving deeper (subcortical and medullar) tissue layers unprotected against cryodamage [26]. We tried to extend the level of protection by testing three other cryoprotective agents that were previously used in analogous studies [30, 31] and comparing them with DMSO. As shown in Fig. 1, freezing a bovine ovary without any protective measures diminished its glucose uptake in the 4 days of culture by more than 80% on all tissue levels (condition 1 versus 2). In line with our previous work, submerging the ovary for 15 min in DMSO (condition 3) prior to cryopreservation completely protected the ovarian cortex but did not protect the subcortical and the medullar tissue layers. Substituting DMSO with either propanediol (condition 4) or butanediol (condition 6) did not provide protection on either tissue level. Using ethylene glycol (condition 5) resulted in a varying protection level for the cortical tissue. In four different ovaries, a protection level of between 19 and 93% (mean 59%) was found, which was statistically different from fresh tissue (*P* < 0.001). On the subcortical and medullar layers, no protective effect was observed (Fig. 1, middle and right panels; *P* < 0.001). Unexpectedly, the levels of glucose uptake of subcortical and medullar tissues derived from ovaries treated with ethylene glycol, propanediol, or butanediol were lower than the glucose uptake in tissue frozen and thawed without any protection against cryodamage. However, these differences did not reach statistical significance (*P* > 0.05).

Whereas a submersion time of 15 min is sufficient for DMSO to obtain maximum protection in the cortical layer (Fig. 1, left panel), the other cryoprotective agents might require a longer incubation time to achieve protection. To test this, we repeated the experiment with a submersion period of 3 h. The results were basically identical to the previous experiment, i.e., full protection of cortical tissue by DMSO, some protection of the cortex by ethylene glycol, and no protective effect with the other cryoprotective agents (data not shown).

### Combining submersion with perfusion of whole ovaries increases the protective effect of DMSO and ethylene glycol in the subcortical and medullar tissues

**Fig. 2:**
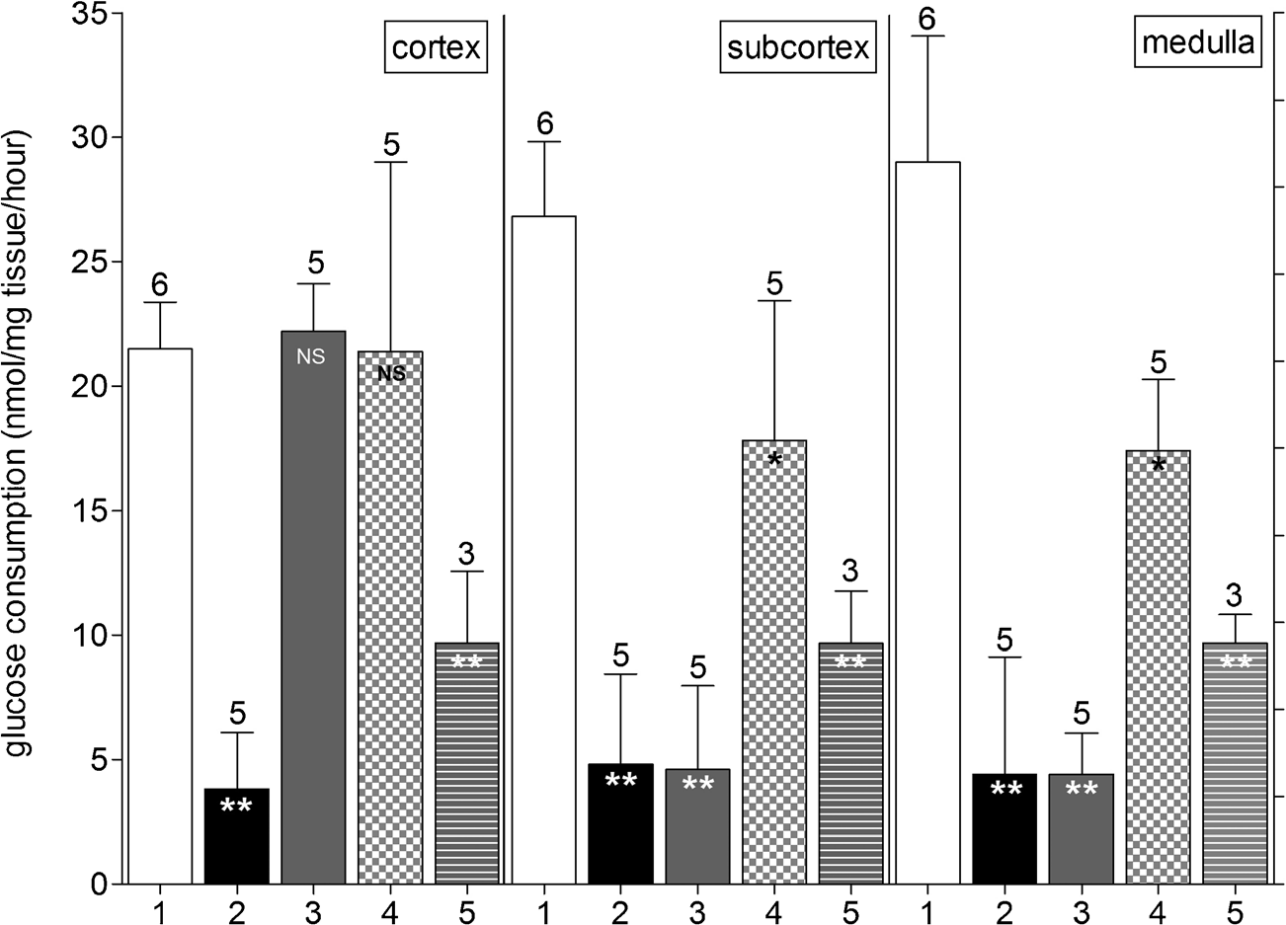
In vitro glucose uptake of ovarian tissue fragments derived from bovine ovaries submerged in, and perfused with, cryoprotectants. Fresh ovaries (condition 1; positive control, *white bars*); ovaries cryopreserved and thawed without any protection (condition 2; negative control, *black bars*); from ovaries submerged in a solution containing 10% DMSO for 15 min prior to cryopreservation (condition 3, *gray bars*). Ovaries submerged in, and perfused with, 10% DMSO for 30 min are shown as condition 4 (*checkered bars*) and ovaries submerged in, and perfused with, 10% ethylene glycol for 30 min are shown as condition 5 (*striped bars*). Tissue fragments were collected from the ovarian cortex (*left panel*), the subcortex (*middle panel*), and the medulla (*right panel*). Shown is the mean ±SD; the *number above bars* represents the number of tested ovaries per condition. Statistically significant differences from glucose uptake by fresh tissue are shown in *bars*; *P < 0.05; **P < 0.001; *NS* no statistical significance. Note the complete protection against cryodamage of cortical fragments derived from ovaries after submersion in and perfusion with DMSO and the partial protection of the subcortex and the medulla (condition 4). With ethylene glycol only, approximately 50% of the protection level obtained with DMSO was observed (condition 5)

In a previous report [26], we have shown that a combination of submerging and perfusion with a solution containing 10% DMSO extended the protective effect to the deeper tissue layers of an intact bovine ovary. In the next set of experiments, we therefore analyzed the protective effect of combining submersion and perfusion with ethylene glycol, using the same treatment with DMSO as a reference (Fig. 2). Submersion in 10% DMSO for only 15 min (condition 3) fully protected the cortical ovarian layer (left panel) while not protecting the subcortex (middle panel) or medulla (right panel). Combining submersion and perfusion in/with DMSO for 30 min (condition 4) still resulted in full protection of the cortex, while only partially protecting the subcortex and medulla for 66 and 60%, respectively. Combining submersion and perfusion with ethylene glycol (condition 5) increased the protection of deeper layers compared with submersion only from 5 to 36% for the subcortex and from 7 to 33% for the medulla. The level of cryoprotection for the subcortex and the medulla with submersion/perfusion with ethylene glycol (condition 5) was considerably less than the protection level achieved by DMSO submersion/perfusion (condition 4). Note that due to the lack of cryoprotection by butanediol or propanediol in the submersion experiments (Fig. 1), these compounds were not further used in the subsequent perfusion experiments shown in Fig. 2.

### Time between removal of the ovary and start of the heparin perfusion is crucial for successful cryopreservation of the entire ovary

**Fig. 3:**
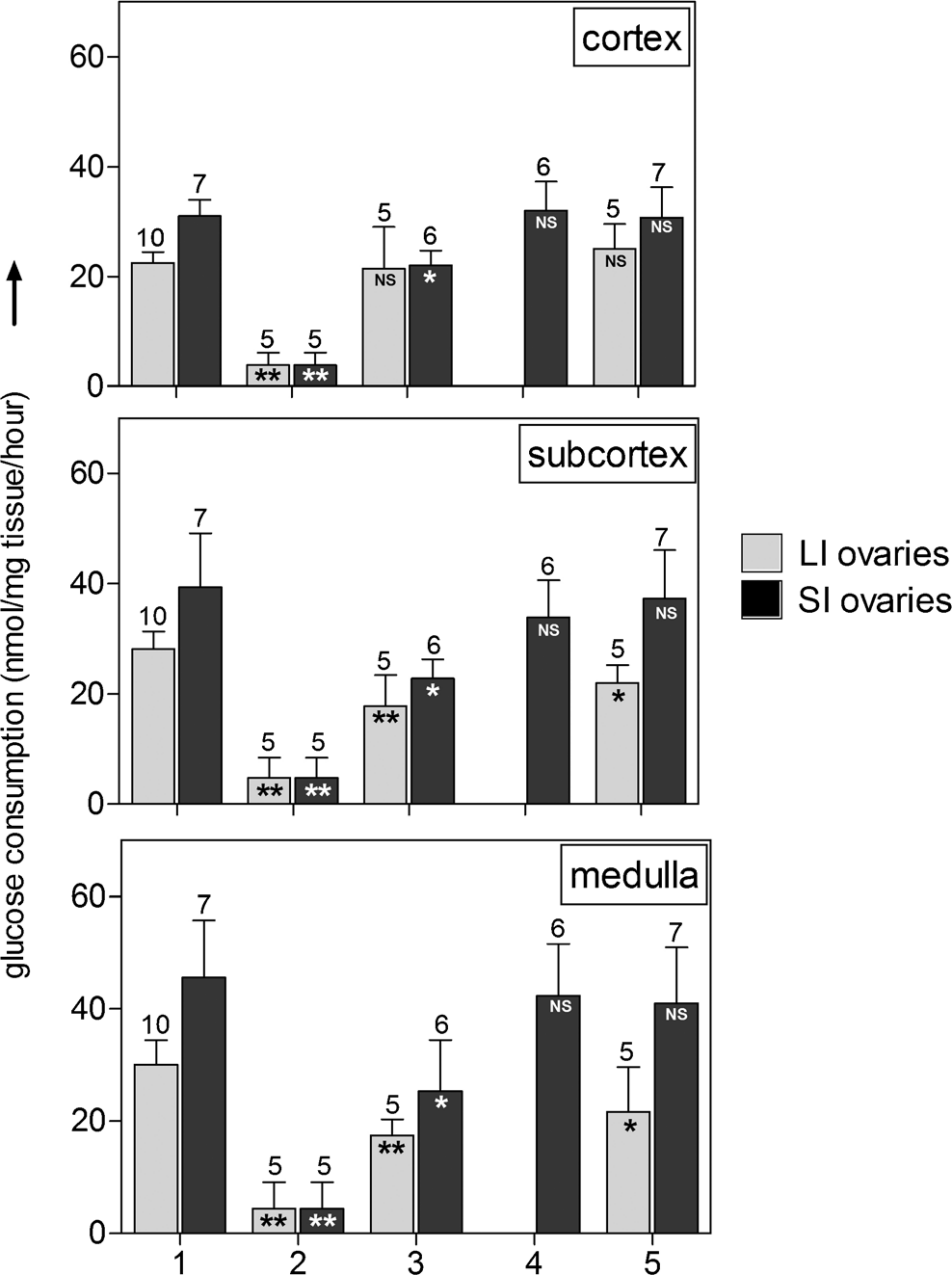
The effect of warm ischemia and length of the perfusion time with cryoprotectant on whole ovary viability. In vitro glucose uptake of ovarian tissue fragments from bovine ovaries derived from animals that were processed (perfused with heparin and transferred to ice) 30–50 min after death (*gray bars*; “long ischemic (LI) ovaries”) versus 15–20 min after death (*black bars*; “short ischemic (SI) ovaries”). Fresh ovaries (condition 1; positive control); ovaries cryopreserved and thawed without any protection (condition 2; negative control); from ovaries submerged in, and perfused with, 10% DMSO for 30 min (condition 3) or 60 min (condition 4) or 120 min (condition 5) prior to cryopreservation. Shown is the mean ±SD; the *number above bars* represents the number of tested ovaries per condition. Statistically significant differences from glucose uptake by fresh tissue are shown in *bars*; *P < 0.05; **P < 0.001; *NS* no statistical significance. Note the increased glucose uptake of fresh SI ovarian tissue compared to fresh LI ovarian tissue (condition 1). Also, the DMSO-mediated protection levels against cryodamage are much higher (>95% protection) in the SI ovarian tissue, compared to LI ovarian tissue (50–70% protection)

From the experiments described above, we concluded that DMSO was superior to the other tested cryoprotective agents and, therefore, the cryoprotectant of choice for further optimization of the cryopreservation process. Even with DMSO, however, we did not succeed in increasing protection levels of the subcortex and the medulla beyond the 66% mark, which was statistically different from fresh tissue (*P* < 0.05; Fig. 2, condition 4 in the middle and right panels). We therefore also investigated the possible negative effect of the relatively long time period of warm ischemia at the abattoir (30–50 min) between the death of the animal and the moment when we could obtain its ovaries for further processing. Eventually, however, we were able to obtain the ovaries 10–15 min after death of the animal, thereby minimizing the period of warm ischemia. With these so-called “short ischemic (SI) ovaries,” we repeated a number of experiments that were previously performed with “long ischemic (LI) ovaries” (i.e., ovaries obtained 30–50 min after death), using DMSO as a cryoprotectant. Results are shown in Fig. 3.

The glucose uptake of fresh SI ovaries (i.e., obtained 10–15 min after death; Fig. 3, condition 1, black bars) was significantly (*P* ≤ 0.0001) higher than the glucose uptake of fresh LI ovaries (i.e., obtained 30–50 min after death; condition 1, gray bars). This was apparent in all three tissue layers. After submerging/perfusing the LI ovaries for 30 or 120 min with 10% DMSO, we observed a completely protected cortex (upper panel, gray bars) and partially protected subcortex (middle panel, gray bars) and medulla (lower panel, gray bars). Although prolonging the incubation from 30 min (condition 3) to 120 min (condition 5) led to a slight increase of the protection level (from 60 to about 75%), complete protection was not achieved and a statistically significant difference with the glucose uptake by fresh tissue remained (*P* < 0.05). After repeating this experiment with SI (black bars) instead of LI (gray bars) ovaries, we observed a partial protection after submerging and perfusion with DMSO after 30 min (condition 3, black bars), but after a 120-min (condition 5, black bars) incubation time, a protection level of over 90% was achieved in all three tissue layers that was statistically not different from the glucose uptake by fresh tissue of SI ovaries (*P* > 0.05). Considering the possible toxic side effects of DMSO, our aim was to keep the incubation time with DMSO as short as possible. We therefore also tested the protective effect of submersion/perfusion on SI ovaries for an intermediate period of 60 min (condition 4, black bars). As shown in fig. 3, the protection levels obtained with this procedure were comparable to a 120-min incubation and statistically not different (*P* > 0.05) from the levels of fresh tissue in all three layers (condition 1).

**Fig. 4:**
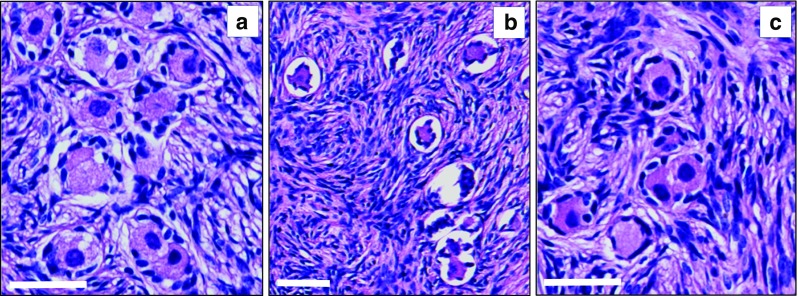
Follicle morphology before and after cryopreservation/thawing of bovine ovaries. Histological hematoxylin and eosin-stained 7-μM sections were prepared from cortical tissue of fresh (**a**) and cryopreserved/thawed bovine ovaries without any cryoprotectant (**b**) and bovine ovaries cryopreserved under optimal conditions (**c**). The vast majority of observed follicles were found to be in the primordial/primary stage of development. In **b**, obvious signs of follicle degeneration can be observed (pyknotic nuclei and granulose cells that are detached from the follicle wall), which are lacking in **a**, **c**. *Bars* represent 50 μM

In bovine ovaries cryopreserved via the most optimal procedure (i.e., the interval between death and start of cryopreservation procedure less than 20 min, and 60 min perfusion/submersion in 10% DMSO), we performed a histological analysis of follicle condition (Fig. 4).

### The optimized DMSO submerging and perfusion protocol also fully protects human ovaries against cryodamage

**Fig. 5:**
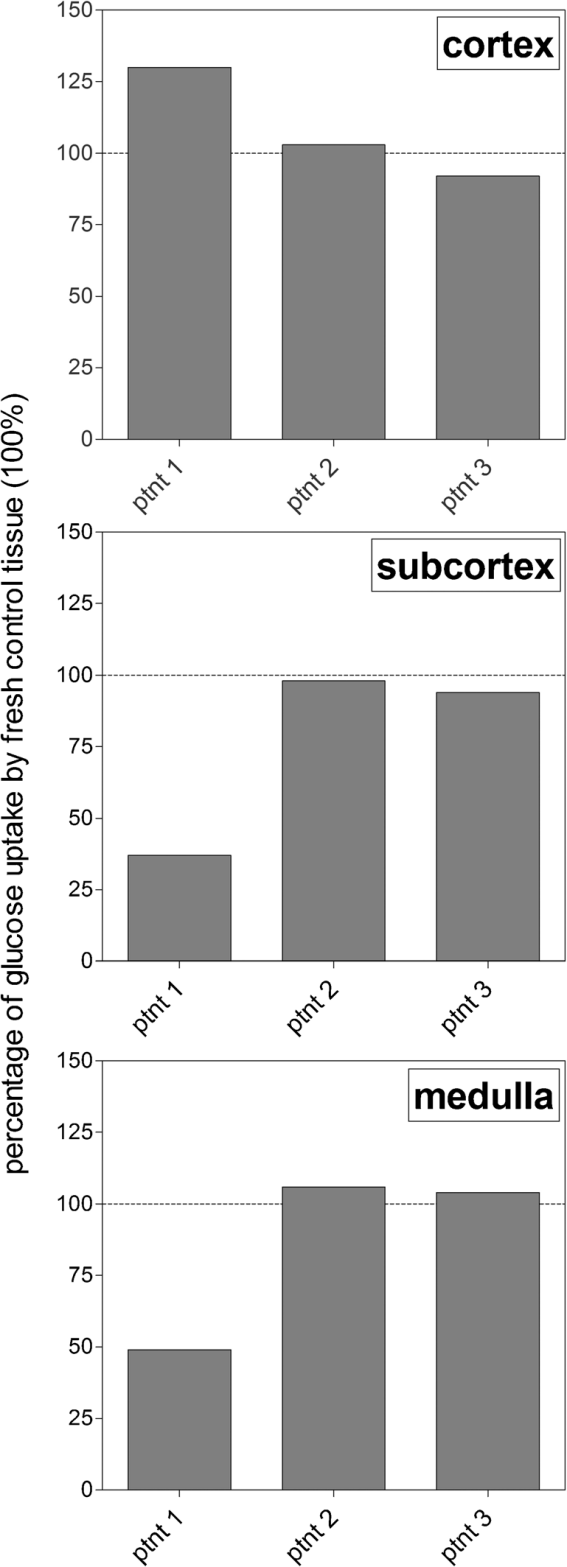
In vitro glucose uptake by fresh and cryopreserved human ovarian tissue. Ovaries were derived from three different patients that had both of their ovaries prophylactically removed. One ovary from each patient was used to prepare fresh tissue fragments (positive control), and the glucose uptake of these fresh fragments was set at 100%. The contralateral ovary of each patient was treated following the optimized cryopreservation protocol by submersion in, and perfusion with, a 10% DMSO solution during 60 min prior to cryopreservation. After cryopreservation/thawing, tissue fragments (of the cortex, subcortex, and medulla) were prepared and cultured. Glucose uptake is expressed as a percentage of the uptake by fresh tissue. Note the complete protection against cryodamage in patients 2 and 3 at all tissue levels, whereas the subcortical and medullar tissue from the suboptimal perfused ovary of patient 1 appeared to have sustained considerable cryodamage, as reflected by the diminished level of glucose uptake

After establishing the optimal procedure to protect intact bovine ovaries against cryodamage during cryopreservation, we cryopreserved intact ovaries from three women, using the optimal protocol we established in the bovine model system as described above. From each patient, one ovary was used to prepare tissue fragments that were cultured fresh (positive control), whereas the contralateral ovary was perfused with heparin and cryopreserved using a combination of 10% DMSO for submersion and perfusion for 60 min. Considering the importance of starting the perfusion with heparin as soon as possible after obtaining the ovary, this perfusion was performed in the operation room, only minutes after the ovaries had been surgically removed. As judged by the blue coloration of the ovary during the perfusion with heparin/methylene blue, the perfusion of the ovary used for cryopreservation of patient 1 was not completely successful, as only part of the ovary turned blue. The ovaries that were to be cryopreserved from patients 2 and 3, on the other hand, turned completely blue shortly after start of the perfusion, indicating that the perfusion procedure had been successful. In Fig. 5, the glucose uptake of the tissue fragments of the fresh (non-cryopreserved) ovaries was set at 100% to enable inter-patient comparisons. In patient 1, the cortical layer of the ovary was fully protected against cryodamage (upper panel, left bar), whereas in the subcortex (middle panel, left bar) and the medulla (bottom panel, left bar), protection levels of only 30–40% were achieved. In patients 2 and 3, on the other hand, all three ovarian tissue layers were protected for 90–100% by applying our optimized cryopreservation protocol.

### The cryopreservation procedure causes no visible damage to either follicles or the vascular endothelium in the human ovarian tissue

**Fig. 6:**
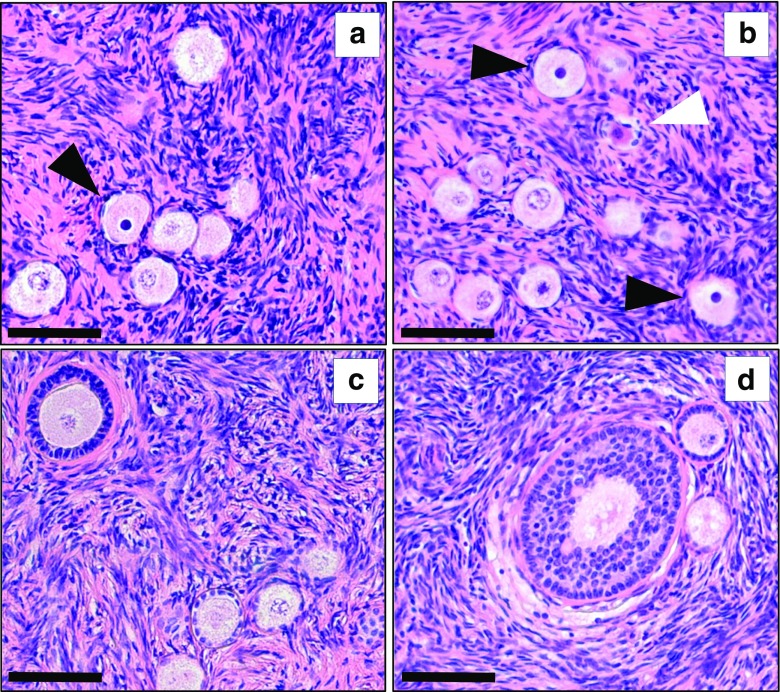
Follicle morphology before and after cryopreservation/thawing of human ovaries. Histological hematoxylin and eosin-stained 7-μM sections were prepared from cortical tissue of fresh (**a**, **c**) and cryopreserved/thawed human ovaries (**b**, **d**). In **a**, **b**, primordial follicles with pyknotic nuclei (*black arrowheads*) and cytoplasmic shrinkage/detached granulose cells (*white arrowhead*) are indicated. In **c**, **d**, more advanced stages of follicular development are shown next to primordial follicles. *Bars* represent 100 μM

We counted the number of follicles in 15 hematoxylin and eosin-stained sections (100 μm apart) from the fresh, non-cryopreserved ovaries of the three patients. It should be noted that, as all three patients were of relatively advanced age, the number of observed follicles was limited. We therefore decided to count all follicles in the 15 tissue sections, rather than a fixed number for each patient (number of counted follicles is shown in Table 4). Our results showed that more than 90% of the follicles were morphologically normal, whereas less than 10% showed cytoplasm shrinkage, disorganized granulosa cells, or pyknotic nuclei as a sign of follicle degeneration (Fig. 6, Table 4). After cryopreservation and thawing of the suboptimal perfused ovary of patient 1, the percentage of morphologically normal primordial/primary follicles was 81%, whereas the optimally perfused ovaries of patient 2 and patient 3 contained 96 and 90% morphologically normal primordial/primary follicles, respectively. Of the follicles observed (*n* = 613), 95% was at the primordial or primary stage.

**Table 4:** Percentage of morphologically normal follicles in cortical fragments of control ovaries and the contralateral cryopreserved/thawed ovaries of three patients. The number of degenerated follicles and the total number of counted follicles are indicated. Follicles were classified according to Gougeon [27]

	Primordial	Primary	Secondary	Antral
Patient 1				
Control ovary	89% (4/34)	94% (1/16)	100% (0/1)	0/0
Cryopreserved ovary	77% (15/50)	97% (1/34)	95% (1/19)	100% (0/3)
Patient 2				
Control ovary	98% (2/105)	100% (0/19)	100% (0/3)	0/0
Cryopreserved ovary	96% (5/137)	100% (0/2)	0/0	0/0
Patient 3				
Control ovary	100% (0/64)	100% (0/8)	100% (0/2)	0/0
Cryopreserved ovary	90% (11/109)	100% (0/6)	100% (0/1)	0/0

**Fig. 7:**
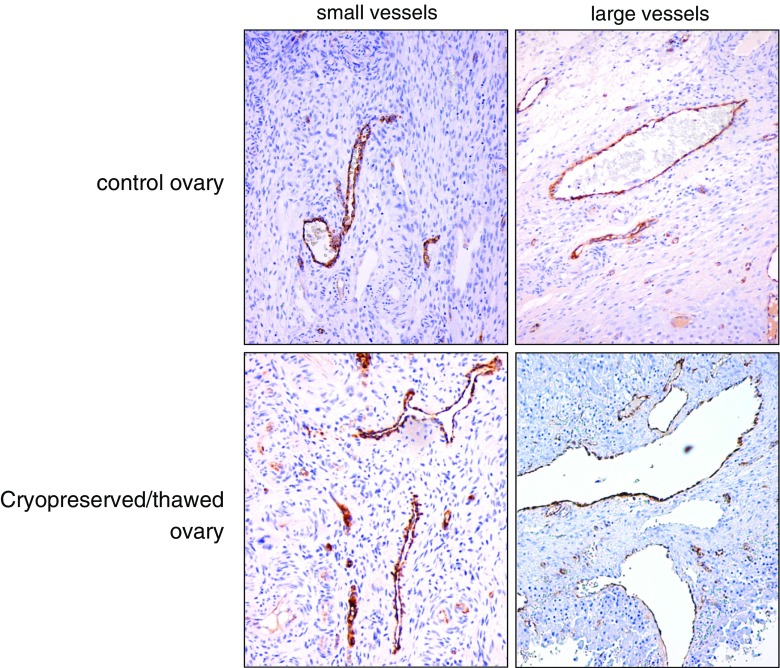
Fresh and cryopreserved human ovarian tissue stained immunohistochemically with anti-human factor VIII antibody to highlight endothelial cells. No apparent damage to the vascular endothelial cell layer is visible in the cryopreserved/thawed ovaries. Note the presence of erythrocytes in the vessels of the fresh tissue, which are lacking in the perfused cryopreserved ovarian tissue

Damage to the vascular component of the ovary could be a side effect of a prolonged incubation time with DMSO [32]. To assess damage to the endothelium, we performed immunohistochemical staining with anti-human factor VIII antibody in fresh as well as cryopreserved and thawed human ovarian tissue. As shown in Fig. 7, no detached endothelial cells or other apparent damage to the endothelial cell lining was visible.

## Discussion

In this paper, we present a procedure to cryopreserve and thaw an intact ovary without apparent cryodamage. We found it was crucial to confine the period of warm ischemia to an absolute minimum. Submersion in, and perfusion with, 10% DMSO for 60 min was required for a complete protection against cryodamage on all tissue levels.

A comparable perfusion time (40 min) required for optimal cryopreservation of bovine ovaries with DMSO was reported by Zhang et al. [33, 34]. While the majority of our data was obtained using bovine ovaries as a model system, our final experiments with human ovaries show that our bovine data actually can be extrapolated to the human situation.

We have shown that whereas the vasculature of two human ovaries was colored blue in their entirety by the methylene blue/heparin perfusion fluid, the vasculature of the third ovary was only partly colored. This observation was in accordance with the less successful cryopreservation (based on its glucose uptake) of the latter ovary. Most likely, clot formation had occurred in this ovary, thereby not only preventing the heparin/methylene blue solution to completely perfuse the ovary but also hindering the subsequent perfusion with the cryoprotectant solution. Preventing clotting is an important factor in not only the cryopreservation procedure but also post autotransplantation [20]. Clearly, a functional vascular bed of the ovary is evidently a prerequisite for several crucial steps in the cryopreservation/thawing and autotransplantation procedure.

Another factor in our model system which proved to be essential for successful cryopreservation was the duration of the warm ischemic period between death of the animal and cooling of the tissue. By reducing this period of time as much as technically possible, the glucose uptake of the fresh tissue increased considerably. In addition, submersion in, and perfusion with, DMSO resulted in full protection of the glucose uptake of this less ischemic tissue after cryopreservation and thawing. When surgically removing a human ovary for cryopreservation, there should be no time loss between removal of the ovary from its vascular pedicle and start of the perfusion in order to keep the warm ischemic period as short as possible. The detached ovary should obviously not be left in the abdominal cavity during prolonged surgery but should be transferred to cold (0 °C) medium without delay. In addition, the level of experience of the person performing the perfusion procedure is an important factor in the efficacy and efficiency of intact ovary perfusion [35].

As far as the choice of the cryoprotectant is concerned, our results indicate that butanediol and propanediol should not be used. While we observed some cryoprotective effect of ethylene glycol, in our study, DMSO proved to be clearly superior. In two large European centers for cryopreservation of human ovarian cortex strips, both these cryoprotectants are being used [36, 37]. Both centers have reported several pregnancies after autotransplantation of ovarian tissue fragments [15, 38, 39], indicating that they are both successful in cryopreserving human ovarian tissue. The relative lack of protection against cryodamage which we observed with ethylene glycol may be explained by the fact that we cryopreserved intact ovaries rather than isolated ovarian cortex strips.

Our findings are predominantly based on our glucose uptake assay as a readout system for cryodamage [26]. This assay has been successfully used to quantify cryodamage in ovarian tissue in several other studies [40–43]. As stromal cells comprise more than 90% of the ovarian volume, it is safe to say that this assay predominantly provides information on the viability of the stromal cell compartment. Assays that combine the assessment of glucose uptake with histology, such as the methylthiazolyl blue tetrazolium (MTT) assay described by Torre et al. [35, 44], may provide valuable additional information on the condition and viability of the ovarian tissue.

Obviously, the condition of the follicles, the internal ovarian vasculature, and the vascular pedicle will also have to be assessed before cryopreservation and, consecutively, autotransplantation of an intact human ovary can be performed. Although immunohistochemical evaluation of the integrity of the vascular endothelial lining of both large and small blood vessels did not show any gross irregularities in human cryopreserved ovaries, morphological assessment alone is most probably not sufficient. In previous reports, a number of techniques have been used to demonstrate that not only the cryopreservation process itself but also the perfusion technique that is used to saturate the intact ovine ovaries with cryoprotectant, inflicted damage on several levels [32, 45, 46]. Evidence of vascular damage in cryopreserved ovine ovaries was demonstrated by Onions et al. [32], by the extravasation of fluorescent microspheres, and the presence of clots in cryopreserved ovaries. However, a recent report on the successful cryopreservation and autotransplantation of intact sheep ovaries leading to restoration of ovarian function and birth of normal offspring used DMSO as a cryoprotectant [20] indicates that despite its potential toxic side effects, DMSO can actually be used to successfully cryopreserve intact ovaries of a large mammal.

The possible toxicity of DMSO may be considered a contraindication for the use of this cryoprotectant in the cryopreservation of human cells, tissue fragment, and in our case, intact organs. Although toxicity of DMSO has indeed been demonstrated in a number of instances, there are now also data available indicating that DMSO toxicity may not be a major factor in the cryopreservation of (human) reproductive tissue. DMSO is widely used in the cryopreservation of human surplus embryos derived from IVF procedures. In addition, the majority of children that were born after autotransplantation of ovarian cortical tissue were derived from tissue that was cryopreserved using DMSO as a cryoprotectant. Should, however, major issues concerning the use DMSO arise in the future, the use of less or even non-toxic alternatives such as trehalose should be further investigated [47].

The transplantation of a non-cryopreserved, fresh intact human ovary is, in fact, technically possible as shown by Silber and Gosden [48] and Silber et al. [49], who performed this procedure on identical twin sisters, resulting in healthy offspring. When autotransplanting frozen/thawed intact ovaries derived from cancer patients, however, there are two additional important issues that should be addressed.

First, tumor cells, possibly leading to reintroduction of the malignancy after autotransplantation, may be present in the ovarian tissue. The number of metastasized tumor cells is very likely related to the tissue volume that is autotransplanted. Consequently, autotransplanting a whole ovary poses a much higher risk compared to the much smaller ovarian cortical strips. Whole ovarian autotransplantation should therefore only be offered to cancer patients when ovarian involvement can be virtually excluded. In a systematic review on the chance of different tumor types metastasizing in the ovary we performed [50], we show that information regarding the metastasizing capacity of most types of primary tumors is either incomplete or lacking completely. We therefore recommend that until our knowledge on this subject has been improved, whole ovary autotransplantation can only be carried out safely on benign indication.

Secondly, the inherent risk with cryopreserving and autotransplanting a whole ovary is that any calamity that may occur during the procedure results in the loss of all the oocytes that are present in the ovary. Clot formation during the autotransplantation is, in this respect, the most prominent risk. In clinical practice, therefore, we suggest that for the time being, whole ovary autotransplantation should be offered only to patients with a very high chance of becoming sterile due to their treatment. One whole ovary should then be cryopreserved, whereas a large biopsy can be obtained from the ovary that is left in situ to obtain tissue strips. These cortex strips may be used as a backup strategy, should the autotransplantation of the whole ovary fail.

In this study, we identified a number of crucial parameters for optimizing the quality of cryopreserved and subsequently thawed (human) intact ovaries. These include the time between surgical removal of the ovary and start of the cryopreservation process (as short as possible), the optimal cryoprotectant (DMSO), the preferred technique to administer the cryoprotectant to the intact ovary (combining perfusion with submersion), and finally, the optimal perfusion/submersion time in DMSO (60 min).

In conclusion, whole ovarian autotransplantation in humans still has a number of obstacles to overcome. Nevertheless, our optimized cryopreservation procedure represents an important step in introducing whole ovary autotransplantation in clinically applied fertility preservation.
